# Human ISWI complexes are targeted by SMARCA5 ATPase and SLIDE domains to help resolve lesion-stalled transcription

**DOI:** 10.1093/nar/gku565

**Published:** 2014-07-02

**Authors:** Özge Z. Aydin, Jurgen A. Marteijn, Cristina Ribeiro-Silva, Aida Rodríguez López, Nils Wijgers, Godelieve Smeenk, Haico van Attikum, Raymond A. Poot, Wim Vermeulen, Hannes Lans

**Affiliations:** 1 Department of Genetics, Medical Genetics Cluster, Cancer Genomics Netherlands, Erasmus MC, Rotterdam, 3015 GE, The Netherlands; 2 Department of Toxicogenetics, Leiden University Medical Center, Leiden, 2333 ZC, The Netherlands; 3 Department of Cell Biology, Medical Genetics Cluster, Erasmus MC, Rotterdam, 3015 GE, The Netherlands

## Abstract

Chromatin compaction of deoxyribonucleic acid (DNA) presents a major challenge to the detection and removal of DNA damage. Helix-distorting DNA lesions that block transcription are specifically repaired by transcription-coupled nucleotide excision repair, which is initiated by binding of the CSB protein to lesion-stalled RNA polymerase II. Using live cell imaging, we identify a novel function for two distinct mammalian ISWI adenosine triphosphate (ATP)-dependent chromatin remodeling complexes in resolving lesion-stalled transcription. Human ISWI isoform SMARCA5/SNF2H and its binding partners ACF1 and WSTF are rapidly recruited to UV-C induced DNA damage to specifically facilitate CSB binding and to promote transcription recovery. SMARCA5 targeting to UV-C damage depends on transcription and histone modifications and requires functional SWI2/SNF2-ATPase and SLIDE domains. After initial recruitment to UV damage, SMARCA5 re-localizes away from the center of DNA damage, requiring its HAND domain. Our studies support a model in which SMARCA5 targeting to DNA damage-stalled transcription sites is controlled by an ATP-hydrolysis-dependent scanning and proofreading mechanism, highlighting how SWI2/SNF2 chromatin remodelers identify and bind nucleosomes containing damaged DNA.

## INTRODUCTION

Deoxyribonucleic acid (DNA) is continuously damaged by environmental agents and endogenous factors. DNA damage interferes with transcription and replication, causing cell death, chromosomal aberrations or mutations, eventually leading to aging and tumorigenesis (1). To protect against the adverse effects of DNA damage, organisms are equipped with diverse DNA repair and associated DNA damage signaling pathways, collectively called the DNA damage response (DDR) (2).

Nucleotide excision repair (NER) removes different types of helix-distorting DNA lesions, including ultraviolet (UV)-induced cyclobutane pyrimidine dimers (CPDs) and pyrimidine (6-4) pyrimidone photoproducts (6-4PPs). Its biological relevance is illustrated by the severe cancer prone and/or progeroid features presented by patients suffering from rare hereditary NER-deficient syndromes (1). NER consists of two damage recognition pathways: global genome repair (GG-NER) and transcription-coupled repair (TC-NER). GG-NER detects lesions located anywhere in the genome and is initiated through cooperative damage detection by the UV-DDB and XPC/HR23B protein complexes (3). TC-NER repairs transcription blocking damage and is initiated by ribonucleic acid (RNA) Polymerase II (RNApolII) stalling at lesions, which attracts the essential TC-NER factors Cockayne Syndrome A (CSA) and Cockayne Syndrome B (CSB) and the UVSSA/USP7 complex (4). Damage recognition leads to the recruitment of the transcription factor IIH to verify the damage and open the surrounding DNA helix. Next, Xeroderma Pigmentosum group A (XPA) and Replication Protein A (RPA) bind to stabilize the repair complex and properly orient the structure-specific endonucleases XPF/ERCC1 and XPG, which excise the damaged strand. The resulting 30 nucleotide single strand DNA gap is filled and sealed by DNA synthesis and ligation (5).

Chromatin interferes with DNA binding of proteins implicated in DNA-transacting processes such as transcription, replication and DNA repair. For efficient execution of these processes, chromatin is commonly modified to regulate the access of proteins to DNA. Adenosine triphosphate (ATP)-dependent chromatin remodeling complexes modify chromatin by catalyzing the disruption of DNA–histone contacts and can slide or evict nucleosomes or alter their composition (6). Four structurally related conserved families of ATP-dependent chromatin remodeling complexes have been described: SWItching defective/Sucrose NonFermenting (SWI/SNF), INOsitol requiring 80 (INO80), Chromodomain Helicase DNA binding (CHD) and Imitation SWItch (ISWI). Next to their established roles in transcription and replication, it has recently become clear that these remodeling complexes are also implicated in the DDR, including NER (7,8). The mammalian and yeast SWI/SNF adenosine triphosphatase (ATPase) BRG1 and several regulatory subunits interact with GG-NER initiation factors Xeroderma Pigmentosum group C (XPC) or Damage-specific DNA Binding protein 2 (DDB2) and stimulate efficient repair of CPDs (9–12). INO80 was also found to play a role in GG-NER, in yeast to restore repair-induced nucleosome loss (13) and in mammals to regulate XPC recruitment and efficiency of repair (14). It was speculated that chromatin compaction may not be a major hurdle for TC-NER as chromatin is already opened because of transcription (15). However, several chromatin modifying factors have been linked to this process as well. The histone acetyl-transferase p300 and HMGN1 were found to associate with TC-NER complexes in a CSB-dependent manner (16). In addition, efficient restart of transcription after TC-NER was found to depend on histone methyltransferase DOT1L (17) and on accelerated histone H2A exchange and new histone H3.3 deposition, mediated by the FACT and HIRA histone chaperones, respectively (18,19). Furthermore, the TC-NER key factor CSB exhibits ATP-dependent chromatin remodeling activity *in vitro* (20,21), which is stimulated by the histone chaperones NAP1L1 and NAP1L4 (22). Although in *Caenorhabditis elegans* (23) as well as in yeast (24) ATP-dependent chromatin remodeling was suggested to promote TC-NER, it is still unknown whether and how ATP-dependent chromatin remodeling plays a role in mammalian TC-NER.

Using genetic screening in the nematode *C. elegans* to find novel chromatin-associated proteins involved in the UV-induced DDR, we have previously identified *isw-1* (23). *isw-1* is orthologous to mammalian SNF2H/SMARCA5 (25), the major catalytic ATPase subunit of several ISWI-type chromatin remodeling complexes (26), suggesting that these complexes play an important role in the cellular response to UV-induced DNA damage. Here, we used a live cell imaging approach to identify a new function for SMARCA5 and its binding partners Williams Syndrome Transcription Factor (WSTF) and ATP-utilizing Chromatin assembly and remodeling Factor 1 (ACF1) in mammalian TC-NER, which is mechanistically distinct from its role in response to double strand DNA breaks (DSBs) (27–32). Our findings indicate that ISWI chromatin remodeling complexes utilize ATP hydrolysis and the SMARCA5 SLIDE domain to associate with UV-damaged chromatin to specifically promote CSB recruitment and to resolve damage-stalled transcription.

## MATERIALS AND METHODS

### Cell culture

U2OS, HeLa, MRC5, TA24 (UVSSA deficient), XP4PA (XPC deficient) and CS1AN (CSB deficient) cell lines were cultured in a 1:1 mixture of Ham's F10 (Lonza) and Dulbecco's modified Eagle's medium (Lonza) supplemented with antibiotics and 10% fetal calf serum (FCS) at 37°C, 20% O_2_ and 5% CO_2_. Primary wild-type human C5RO fibroblasts were cultured in Ham's F10 (Lonza) supplemented with antibiotics and 15% FCS. U2OS and MRC5 cells expressing Green Fluorescent Protein (GFP)-fusion proteins were generated by transfection and isolation of stable colonies and fluorescence activated cell sorting. To inhibit transcription, cells were treated with α-amanitin (25 μg/ml) for 12 h or with DRB (75 μM) for 1 h. To inhibit methylation, cells were treated with AdOX (adenosine dialdehyde) (20 μM or 100 μM) for 12 h. For poly(ADP-ribose) polymerase (PARP) inhibition experiments, cells were treated with olaparib (AZD2281, 10 μM) or PJ34 (10 μM) for 1 h (29,33). Efficient PARP inhibition by both inhibitors (Supplementary Figure S4B) was demonstrated by immunofluorescence (IF) using monoclonal PAR antibody 10H (Alexis Biochemicals) following 5-min 50-mM H_2_O_2_ treatment, which induces granular nuclear polyADP ribose (PAR) staining (19,34). To inhibit deacetylation, cells were treated with histone deacetylase (HDAC) inhibitors, TSA (trichostatin A, 45 nM) for 20 h or NaBu (sodium butyrate, 10 mM) for 2 h.

### Plasmids and siRNAs

Cloning details for SMARCA5-GFP, ACF1-GFP, GFP-WSTF and CPD-photolyase-mCherry are available upon request. TA24 cells expressing UVSSA-GFP (35) and CS1AN cells expressing GFP-HA-CSB (36) were described before. Site-directed mutagenesis was used to generate the ATPase inactivating SMARCA5 mutant, by changing Lys211 in the nucleotide-binding motif to Arg (37), and to generate SMARCA5 deletion mutants of the HAND (aa 743-843), SANT (aa 741-890) and SLIDE (aa 898-1012) domains. To stably knock down protein expression, cells were transduced with MISSION shRNA (Sigma-Aldrich; Clone ID SHC002 for control; TRCN0000016776 for CSB; TRCN0000083194 for XPA; TRCN0000013217 for SMARCA5; TRCN0000013342 for WSTF), by lentiviral transduction (38) and selection with puromycin. Transient siRNA-mediated knock-down was achieved using Lipofectamine RNAiMAX (Invitrogen) transfection according to the manufacturer's instruction. siRNAs used were from Dharmacon: control (D-001210-05), SMARCA5 (L-011478-00), ACF1 (L-006941-00 and J-006941-05), CSB (L-004888-00), WSTF (custom, AAGCCCGCUUGGAAAGGUACA) and XPC (custom, CUGGAGUUUGAGACAUAUCUU).

### Colony survival

To determine colony survival, ∼300 cells were plated in 6-well plates in triplicate. After 12–16 h, cells were irradiated with a single dose of UV irradiation (0–8 J/m^2^; 254 nm; Philips TUV lamp). After 7 days, colonies were fixed and stained with 0.1% Brilliant Blue R (Sigma) and counted. The survival was plotted as the mean percentage of colonies obtained after treatment compared to the mean number of colonies from the non-irradiated samples.

### UV-induced unscheduled DNA synthesis and recovery of RNA synthesis

Unscheduled DNA synthesis (UDS) was measured following UV-C irradiation (16 J/m^2^) of C5RO primary fibroblasts grown on 24-mm cover slips and transfected with siRNA. Irradiated cells were incubated for 2 h in the presence of 0.1-mM 5-ethynyl-29-deoxyuridine (EdU; Invitrogen) after UV irradiation. Recovery of RNA synthesis (RRS) was performed in siRNA-transfected HeLa or U20S cells 16 h after UV-C irradiation. Unirradiated and irradiated cells were incubated for 2 h in the presence of 0.1-mM 5-ethynyl-uridine (EU). EdU and EU incorporation was visualized using Click-iT Alexa Fluor 594 according to the manufacturer's protocol (Invitrogen). UDS and RRS levels were quantified by measuring and averaging fluorescence intensities for >100 cells with ImageJ software of images obtained with a Zeiss LSM700 confocal microscope.

### IF and western blotting

For IF, cells were grown on 24-mm cover slips for 3 days prior to the experiments and fixed using 2% paraformaldehyde in the presence of 0.1% Triton X-100. Cells were immunostained as described previously (39) and embedded in Vectashield mounting medium with 4',6-diamidino-2-phenylindole (DAPI;Vector Laboratories, Burlingame, CA, USA). Images (Supplementary Figures S2C and S4B) were obtained using an LSM510 META confocal microscope (Carl Zeiss, Inc.). Antibodies used for IF and western blotting were: anti-SMARCA5 (SNF2H; Abcam), anti-CSB (E18, SantaCruz), anti-ACF1 (Novus) and anti-WSTF [affinity purified as described in (40)], anti-CPD (TDM-2; MBL International).

### Live cell confocal laser-scanning microscopy

All live cell confocal laser-scanning images were obtained at 37°C using a Leica TCS SP5 microscope (with Leica Application Suite), except Supplementary Figures S3C and S4D, which were obtained using a Zeiss LSM 510 (with LSM image browser), both equipped with a 100× quartz objective. Local UV-C damage was induced by laser irradiation at 266 nm (Rapp OptoElectronic, Hamburg GmbH), which specifically creates CPD and 64-PP DNA lesions that are repaired by NER but no strand breaks, as described previously (41). To quantify the recruitment of SMARCA5 in the center and periphery of a damaged area, we used three different regions of interest (ROIs), one in the middle, one in the periphery and one in an area of the nucleus not exposed to DNA damage (‘outside’) to check for monitor bleaching (Supplementary Figure S3B). All three ROIs were quantified with ImageJ software and curves were normalized to the first data points prior to damage induction. For every curve, at least 10 cells were measured and all results were confirmed by independent duplicate experiments. Statistical difference between curves was determined by one-way analysis of variance comparison of areas under each curve.

### Immunoprecipitation

CSB (Figure 5C), ACF1 and WSTF (Supplementary Figure S5A and B) were immunoprecipitated using chromatin-enriched nuclear extracts from 10 14-cm culture dishes of GFP-CSB expressing CS1AN cells or five 14-cm culture dishes of U2OS cells expressing ACF1-GFP or GFP-WSTF. Cells were collected 20 min (CSB) or 5 min (ACF1/WSTF) after irradiation (20 J/m^2^) by scraping in 3 ml of phosphate buffered saline (PBS) containing protease inhibitor cocktail (Roche), centrifuged for 5 min at 1500 rpm and washed again with PBS. Cells were swollen in 5 x pellet volume of Hepes buffer (10-mM HEPES pH 7.6, 1.5-mM MgCl2, 10-mM KCl, 0.5-mM Dithiothreitol, protease inhibitor cocktail) for 10 min. Nuclei were isolated by douncing cells with a type A pestle and centrifugation at 3000 rpm for 10 min at 4°C. Nuclei were washed and resuspended in 1.5 x pellet volumes of Hepes buffer (100-mM HEPES pH 7.6, 1.5-mM MgCl2, 150-mM NaCl, 25% glycerol, protease inhibitor, 0.5-mM Dithiothreitol) and subsequently dounced using a pestle type B. Next, chromatin was digested with 25-U Microccocal nuclease (MNase; Sigma) for 1 h at 4°C. These conditions were chosen such that DNA was digested to mononucleosome size. The resulting chromatin-enriched nucleoplasmic fraction was cleared from insoluble nuclear material by centrifugation at 15 000 rpm for 15 min. For immunoprecipitation of SMARCA5 mutants (Figure 7C), extracts were prepared by scraping cells from a 14-cm dish in Radioimmunoprecipitation assay buffer (PBS containing 1% NP-40, 0.5% sodium deoxycholate and 0.1% sodium dodecyl sulphate; Roche protease inhibitor cocktail) followed by sonication (to obtain DNA fragments <800 bp) and 16 000 g centrifugation at 4°C for 10 min to remove insoluble material. Extracts were incubated with GFP-trap beads (Chromotek) for 2 h at 4°C. Subsequently, beads were washed four times in Hepes buffer and boiled in Laemmli sample buffer for analysis by western blotting.

**Figure 1.**
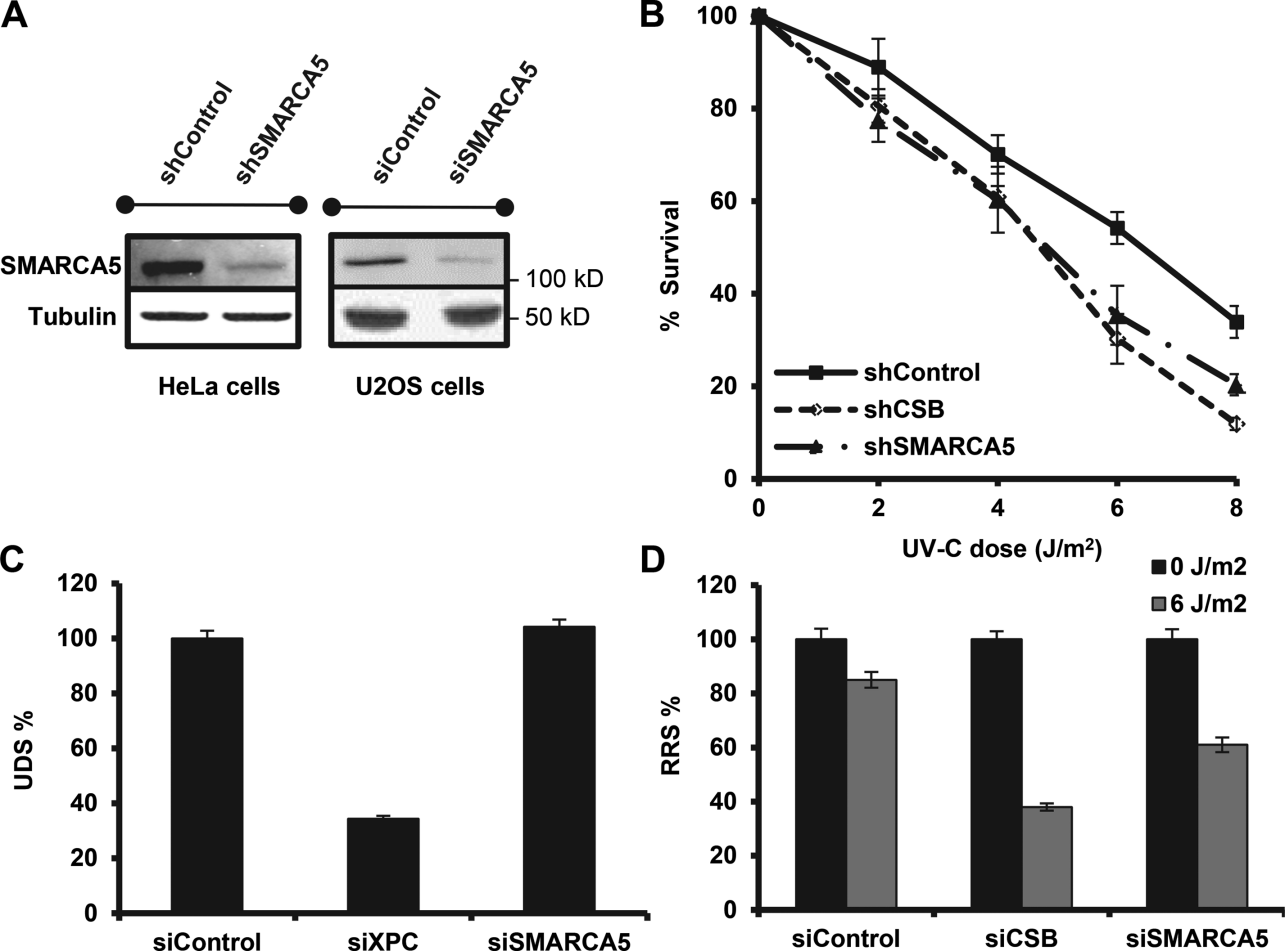
SMARCA5 functions in transcription-coupled repair. (**A**) Immunoblots show reduced SMARCA5 expression levels in HeLa cells stably expressing shRNA and U2OS cells treated with siRNA against SMARCA5. Tubulin was used as loading control. (**B**) SMARCA5 depletion sensitizes cells to UV. Colony survival of HeLa cells stably expressing shRNA against SMARCA5 or CSB following UV irradiation. The percentage of surviving cells is plotted against the applied UV-C dose (J/m^2^). (**C**) DNA repair synthesis (UDS) after UV irradiation (16 J/m^2^), determined by EdU incorporation, as a measure for GG-NER, in wild-type primary fibroblasts (C5RO) (>100 cells for each sample) treated with siRNA. Plotted are, respectively, control (set at 100% UDS), XPC and SMARCA5 siRNAs. (**D**) Recovery of RNA synthesis (RRS), as a measure for TC-NER, determined by EU incorporation 16 h after UV irradiation (0 and 6 J/m^2^) in HeLa cells (>100 cells) treated with, respectively, control (set at 100% at 0 J/m^2^), CSB and SMARCA5 siRNAs. Error bars denote standard error of the mean. The results of all experiments were confirmed at least twice.

**Figure 2.**
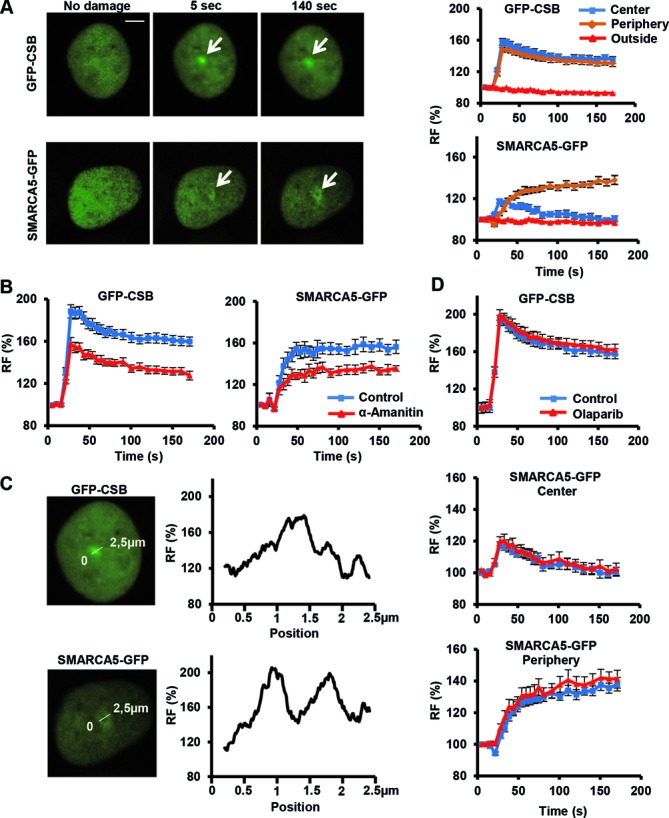
Transcription-dependent SMARCA5 (re)localization to UV-C damage. (**A**) Live cell images (left) before, 5 and 140 s after UV-C (266 nm) laser-induced local damage (arrows) of GFP-CSB (expressed in CSB-deficient CS1AN fibroblasts) and SMARCA5-GFP (expressed in U2OS cells). Scale bar is 5 μm. Graphs (right) show the normalized fluorescence intensities (*n* > 10 cells) that indicate recruitment to the damage center (blue), the damage periphery (orange) and outside the damaged area (red; mean ± standard error of the mean) of GFP-CSB (top) and SMARCA5-GFP. (**B**) Treatment with α-amanitin impairs the binding to DNA damage sites of GFP-CSB (*P* < 0.01 compared to control) and SMARCA5-GFP (peripheral recruitment, *P* = 0.018 compared to control)). (**C**) Line scans of GFP-CSB and SMARCA5-GFP intensity along the indicated line in the image (*n* = 5 cells). (**D**) GFP-CSB (*P* = 0.363 compared to control) and SMARCA5-GFP recruitment (center *P* = 0.682, periphery *P* = 0.36 compared to control) to DNA damage is unaffected upon PARP inhibition using olaparib (*n* > 10 cells, mean ± standard error of the mean). RF denotes ‘relative fluorescence’. All results were confirmed using independent, duplicate experiments.

**Figure 3.**
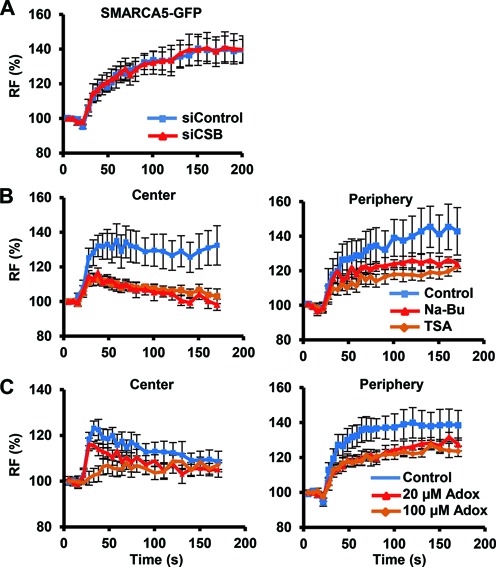
DNA damage association of SMARCA5 requires histone modifications but not NER. (**A**) SMARCA5-GFP recruitment is not affected by siRNA-mediated knock-down of CSB in U2OS cells (*P* = 0.97 compared to control). (**B,C**) SMARCA5-GFP recruitment to DNA damage, both at the center (left) and at the periphery (right), is impaired after HDAC inhibition by TSA (center *P* = 0.006, periphery *P* = 0.040 compared to control) and Na-Bu (center *P* = 0.006, periphery *P* = 0.187 compared to control) treatment (B) and by methyltransferase inhibition using Adox [(C); 100-μM center *P* = 0.064, periphery *P* = 0.041; 20-μM center *P* = 0.197, periphery *P* = 0.076 compared to control]. For each experiment, the mean of *n* > 10 cells ± standard error of the mean is shown. Graphs depict the normalized fluorescence intensity indicating DNA damage recruitment at the damage center or periphery. Results were confirmed using independent, duplicate experiments. RF denotes ‘relative fluorescence’.

**Figure 4.**
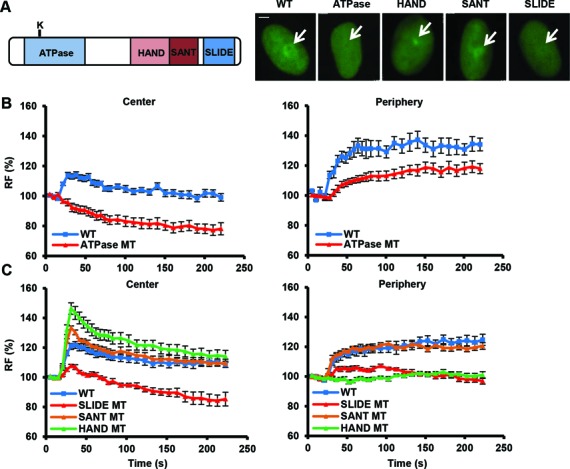
The ATPase, SLIDE and HAND domains of SMARCA5 regulate its damage recruitment. (**A**) Schematic representation of SMARCA5 domains (left). The invariant Lysine 211 in the ATPase domain is indicated by a ‘K’. Representative images (right) show the live cell accumulation pattern of wild type (WT), ATPase-dead, HAND, SANT and SLIDE deletions mutants at local UV-C damage in U2OS cells. Scale bar is 5 μm. (**B**) The SMARCA5 ATPase mutant shows impaired recruitment to DNA damage. Graph of the normalized fluorescence intensity of wild type and ATPase mutant (MT) at the damage center (left) and periphery (right; mean ± standard error of the mean; *n* > 10 cells). (**C**) Graphs of the normalized fluorescence intensity of wild type (WT), HAND, SANT and SLIDE deletion mutants (MT) at the damage center (left) and periphery (right; mean ± standard error of the mean; *n* > 10 cells). Recruitment of the SLIDE domain mutant is impaired, whereas the HAND domain mutant is only recruited to the center of damage. All results were confirmed using independent, duplicate experiments. RF denotes ‘relative fluorescence’.

**Figure 5.**
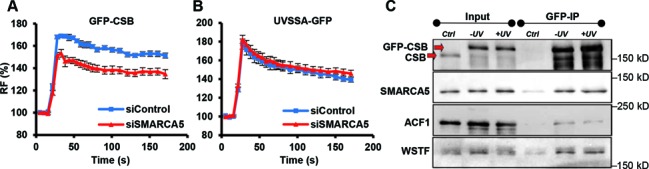
SMARCA5 interacts with CSB and regulates its recruitment. (**A, B**) Graphs of the normalized fluorescence intensity indicating local UV-C-laser-induced DNA damage recruitment of (A) GFP-CSB (*P* < 0.01 compared to control) and (B) UVSSA-GFP (*P* = 0.513 compared to control) in cells siRNA depleted for SMARCA5. *n* > 10 cells, error bars denote standard error of the mean. RF denotes ‘relative fluorescence’. (**C**) GFP immunoprecipitation of GFP-CSB in MNase-treated nuclear extracts shows that SMARCA5, ACF1 and WSTF co-purify with CSB, both in unchallenged conditions (−UV) and 20 min after UV irradiation (+UV). Ctrl is control. All results were confirmed using independent, duplicate experiments.

**Figure 6.**
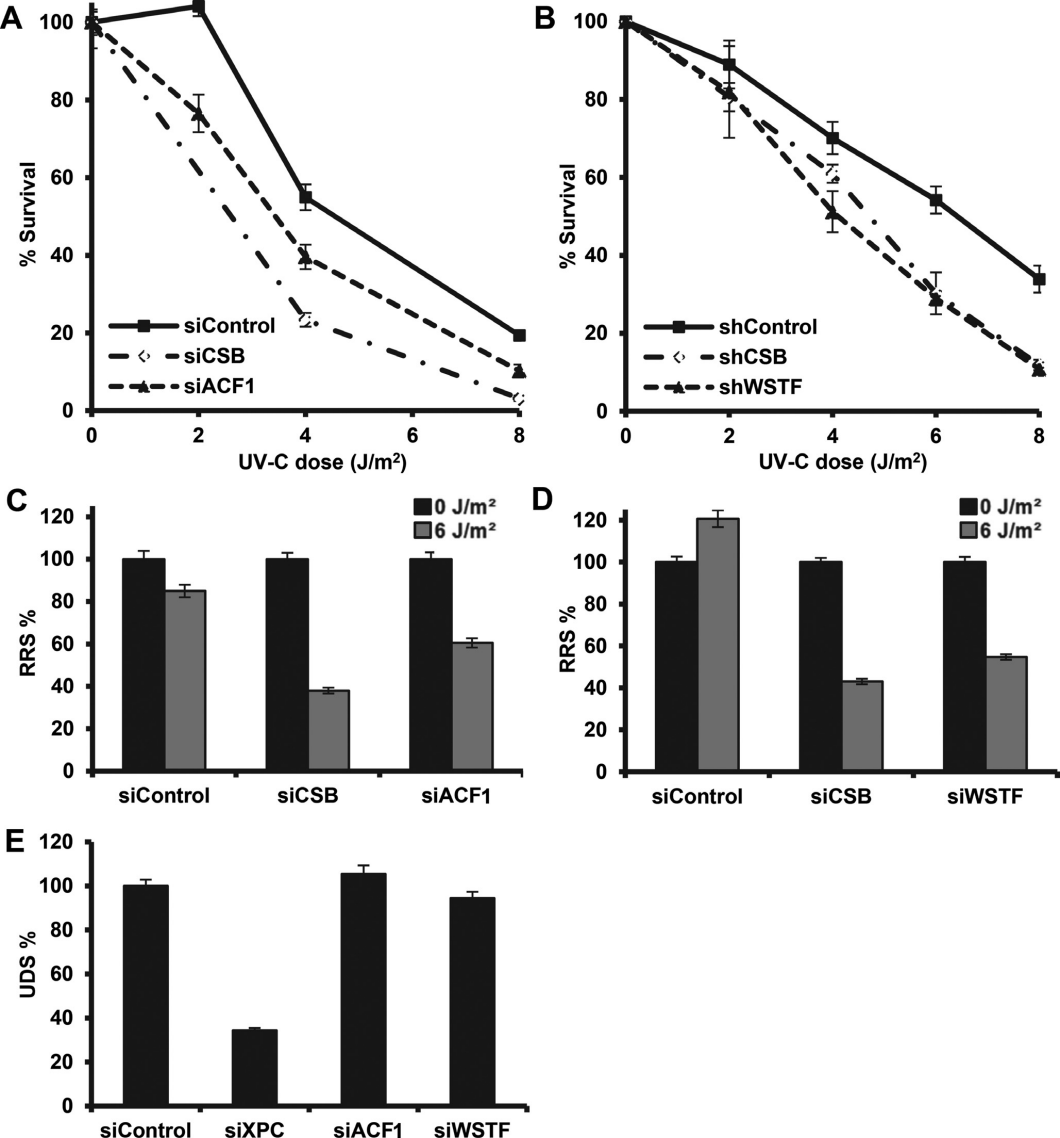
ACF1 and WSTF function in the transcription-coupled response to UV. Depletion of ACF1 and WSTF renders cells hypersensitive to UV and impairs RRS. Colony survival of U2OS cells treated with siRNAs against ACF1 and CSB (**A**) and HeLa cells stably expressing shRNAs against WSTF and CSB (**B**). The percentage of surviving cells is plotted against the applied UV-C dose (J/m^2^). (**C,D**) Impaired RRS, 16 h after 6 J/m^2^ UV-C irradiation, in U2OS cells treated with siRNA against ACF1 or WSTF as measured by EU incorporation. (**E**) siRNA treatment against ACF1 or WSTF in primary C5RO fibroblasts does not affect UDS, as measured by EdU incorporation after 16 J/m^2^ UV-C irradiation. Error bars denote standard error of the mean. All results were confirmed using independent, duplicate experiments.

**Figure 7.**
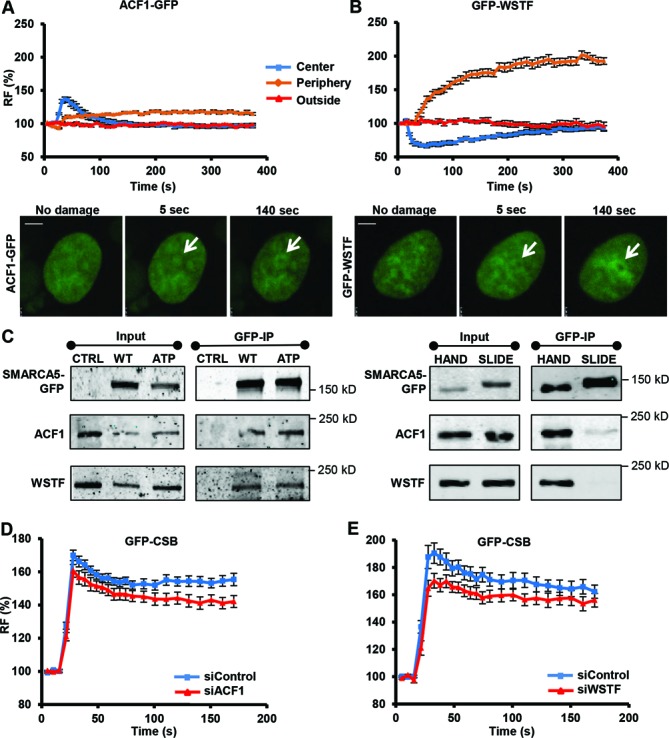
WSTF and ACF1 are recruited to UV damage to regulate CSB recruitment. ACF1-GFP (**A**) and GFP-WSTF (**B**) are recruited to DNA damage induced by UV-C (266 nm) laser. Graphs depict normalized fluorescence intensities indicating DNA damage recruitment in the damage center (blue), the damage periphery (orange) and outside the damaged area (red) (mean ± standard error of the mean; *n* > 10 cells). Representative images of the accumulation of ACF1-GFP and GFP-WSTF at sites of UV damage are shown below the graphs (scale bar is 5 μm). (**C**) GFP immunoprecipitation of GFP-tagged SMARCA5 (WT) and ATPase (ATP), HAND and SLIDE domain deletion mutants. CTRL is control. Only deletion of the SLIDE domain impairs the interaction of SMARCA5 with ACF1 and WSTF. (**D,E**) Graphs of the normalized fluorescence intensity indicating DNA damage recruitment of GFP-CSB in cells in which ACF1 [(D); *P* = 0.033 compared to control] or WSTF [(E); *P* = 0.117 compared to control] is depleted by siRNA (mean ± standard error of the mean; *n* > 10 cells). Results were confirmed using independent, duplicate experiments.

## RESULTS

### SMARCA5 functions in the transcription-coupled response to UV

Following the identification of *isw-1* in the UV response of *C. elegans* (23), we tested whether its mammalian ortholog SMARCA5 is also involved in the UV-DDR. Stable knock-down of SMARCA5 renders HeLa cells hypersensitive to UV, similar to CSB knock-down (Figure 1A and B). These data suggest that SMARCA5 has an evolutionary conserved function in the UV-DDR.

Because chromatin remodeling is thought to be required to facilitate access for NER damage detection proteins (7), we subsequently investigated whether SMARCA5 specifically regulates GG-NER, TC-NER or both. First, we determined UV-induced UDS as a measure of GG-NER (42). Cells treated with siRNA against SMARCA5 (Figure 1A) exhibited a similar UDS level as control treated cells, whereas knock-down of XPC caused a strong UDS reduction (Figure 1C). These data suggest that SMARCA5 is not involved in GG-NER. Next, we measured RRS after UV-induced inhibition, as a measure of TC-NER (42). Cells depleted for both SMARCA5 and CSB showed reduced RRS levels after UV (Figure 1D). Similar results were obtained with an shRNA targeting a different part of the SMARCA5 mRNA, ruling out off-target effects (Supplementary Figure S1A). Importantly, transcription in non-damaged cells was not affected, suggesting that the decrease in RRS is not caused by a general transcription reduction induced by SMARCA5 knock-down (Supplementary Figure S1B). These results indicate that SMARCA5 is specifically involved in TC-NER and/or regulates transcription restart after TC-NER.

### SMARCA5 accumulates at local UV-C damage

TC-NER factors such as CSB localize to DNA damage induced by a 266-nm UV-C laser, which specifically induces CPD and 6-4PP photolesions (Figure 2A and Supplementary Movie S1) (35,41). Stably expressed GFP-tagged SMARCA5 also rapidly accumulated at local UV-C damage, in both U2OS and MRC5 cells (Figure 2A, Supplementary Figure S2A and Supplementary Movie S2), in a dose-dependent manner (Supplementary Figure S2B). This was confirmed by local UV damage induction using a microporous filter (Supplementary Figure S2C) (43). The association of TC-NER factors with TC-NER complexes depends on stalling of RNApolII complexes and thus on active transcription (4,36). Inhibition of RNApolII activity using α-amanitin (44) indeed decreased the accumulation of both CSB and SMARCA5 at local damage (Figure 2B). SMARCA5 recruitment was also attenuated by the transcription elongation inhibitor DRB (Supplementary Figure S3A, in which both recruitment to the center and periphery of the damaged area are quantified as explained below) (45). These results confirm a function of SMARCA5 in TC-NER and suggest that this protein may localize to UV damage depending on RNApolII stalling.

Intriguingly, however, the accumulation characteristics of SMARCA5-GFP were different from those of GFP-CSB. Whereas CSB remained localized in the center of the damage, SMARCA5 swiftly spread from the center to the periphery of the UV-damaged area (Figure 2A, Supplementary Figure S3B and Supplementary Movie S2). Binding kinetics at the periphery were slower but eventually reached a higher level than at the damage center. Fluorescence intensity measurements across the damage confirmed that SMARCA5, but not CSB, re-localized around the damage center (Figure 2C). Furthermore, co-expression of SMARCA5-GFP with mCherry-fused *Potorous tridactylus* CPD-photolyase, which specifically binds to CPDs (46), also showed that SMARCA5 moves away from the area with the highest damage concentration and accumulates at the periphery (Supplementary Figure S3C). This peculiar re-localization was never observed for any of the tested NER proteins (47).

DSB induction by laser micro-irradiation also triggers SMARCA5 recruitment and spreading into adjacent chromatin, which is dependent on PARP activity (29). However, PARP inhibition by olaparib (Figure 2D) or PJ-34 (Supplementary Figure S4A and B) did not affect SMARCA5 recruitment and spreading, indicating that its recruitment and function at UV-induced DNA damage involve a different mechanism than at DSBs. As GFP-CSB recruitment was also unaffected by PARP inhibition (Figure 2D), these results suggest that poly(ADP) ribosylation does not play a role in the assembly of TC-NER factors.

### SMARCA5 recruitment depends on histone modifications but not on NER

DNA damage-induced binding of CSB to stalled RNApolII complexes is essential for the subsequent assembly of most other repair factors (16). Surprisingly, however, depletion of CSB by siRNA did not affect the recruitment of SMARCA5 (Figure 3A). Local damage accumulation of SMARCA-GFP in CSB-deficient CS1AN fibroblasts further confirmed that CSB is not required to bring SMARCA5 to UV-damaged chromatin (Supplementary Figure S4C). SMARCA5 also accumulated normally to UV damage in TC-NER defective UVSSA fibroblasts and GG-NER defective XPC fibroblasts (Supplementary Figure S4C). This suggests that SMARCA5 recruitment does not depend on TC-NER or GG-NER initiation and that SMARCA5 functions upstream or in parallel to TC-NER.

The activity of many ATP-dependent chromatin remodelers is regulated by post-translational modifications of histones. For instance, lysine acetylation of histone H4 N-terminal tails was found to interfere with *Drosophila* and mammalian ISWI/SMARCA5 binding and function (48–50). Increasing histone acetylation using the HDAC inhibitors TSA and NaBu indeed reduced SMARCA5 recruitment to UV damage, both at the center and at the periphery (Figure 3B). Methylation of histone H3 is another post-translational modification that was shown to be necessary for SMARCA5 association with chromatin during transcription and DSB repair (27,51). We found that inhibition of histone methylation using Adox also reduced recruitment of SMARCA5 to UV damage (Figure 3C). Together, these results indicate that chromatin is modified by both histone (de)acetylation and methylation to regulate SMARCA5 localization to UV damage.

### ATPase, SLIDE and HAND domains regulate UV damage-induced accumulation and re-localization of SMARCA5

Next, we determined which SMARCA5 domains are responsible for its damage accumulation and subsequent re-localization. SMARCA5 harbors an ATPase domain at the N-terminus, for ATP hydrolysis, and HAND, SANT and SLIDE domains at the C-terminus (Figure 4A), which were suggested to associate with linker DNA to control nucleosome sliding (52,53). To test involvement of the ATPase domain, we introduced an inactivating mutation by replacing Lys211 in the nucleotide-binding motif with Arg (37). Intriguingly, ATPase dead SMARCA5-GFP did not localize to the center of UV damage and was even depleted from this area (Figure 4A and B). Furthermore, a reduced and delayed recruitment to the periphery of the damage was observed. This suggests that ATP hydrolysis directs SMARCA5 targeting to UV-C-induced DNA damage.

Next, we deleted each of the C-terminal HAND, SANT or SLIDE domains to analyze their involvement. Deletion of the SANT domain did not affect damage binding (Figure 4A and C). Accumulation of the HAND deletion mutant to the center of damage, however, was strikingly higher than wild type and it showed no re-localization to the periphery (Figure 4A and C). In contrast, the SLIDE deletion mutant did not localize to the center of the damage at all, nor to the periphery (Figure 4A and C). Because the SLIDE domain alone is necessary and sufficient for SMARCA5 recruitment to DSBs induced by laser micro-irradiation (28), we also tested UV-C damage recruitment of this domain only. GFP-tagged SLIDE, however, showed a very weak and transient recruitment to UV-C damage, with no re-localization (Supplementary Figure S4D), contrasting the recruitment of this domain to DSBs and that of the whole SMARCA5 protein to UV-C damage. Our results therefore indicate that both the ATPase and SLIDE domains are involved in recognition and binding of SMARCA5 to its nucleosomal target, whereas the HAND domain seems to be involved in SMARCA5 re-localization after initial binding.

### SMARCA5 facilitates CSB binding to UV-induced damage

The UV damage recruitment of SMARCA5 suggests that its chromatin remodeling activity may facilitate access and function of subsequent repair factors. Indeed, as shown in Figure 5A, siRNA-mediated knock-down of SMARCA5 attenuated the recruitment of CSB to local UV damage. Importantly, this reduction was not due to transcription inhibition, because overall transcription levels were similar in cells with and without SMARCA5 (Supplementary Figure S1B). UVSSA recruitment, however, was not dependent on SMARCA5 (Figure 5B), indicating that the effect of SMARCA5 depletion on CSB is not an indirect consequence of changes in chromatin compaction or transcription. Rather, these data point to an important, specific regulatory function of SMARCA5 in TC-NER to facilitate recruitment of CSB, possibly by chromatin remodeling.

Because SMARCA5 regulates CSB, we tested whether both proteins interact by performing native co-immunoprecipitation using nuclear extracts of GFP-CSB expressing cells, which were enriched for chromatin proteins by MNase treatment, and GFP as bait. We found that CSB co-purifies with SMARCA5, in untreated as well as UV-irradiated cells (Figure 5C). This CSB and SMARCA5 interaction, which may be either direct or indirect, confirms a role for SMARCA5 in TC-NER and suggests that both proteins simultaneously promote TC-NER.

### ACF1 and WSTF function to regulate TC-NER

SMARCA5 is the catalytic subunit of several ISWI family ATP-dependent chromatin remodeling complexes including ATP-utilizing Chromatin assembly and remodeling Factor (ACF) (54) and WSTF-ISWI CHromatin remodeling complex (WICH) (55). Both complexes were previously shown to be involved in DSB repair (28,32), while *Drosophila* ACF complex was also shown to facilitate NER of DNA damage in linker DNA *in vitro* (56). The human ACF complex consists of SMARCA5 and ACF1 (57), whereas the WICH complex consists of SMARCA5 and WSTF (55) (Supplementary Figure S5A and B). As shown in Figure 6A and B, knock-down of ACF1 and WSTF (Supplementary Figure S5C) rendered cells hypersensitive to UV. In addition, loss of ACF1 and WSTF clearly reduced RRS (Figure 6C and D) but not UDS (Figure 6E). This was achieved using different siRNAs, ruling out off-target effects (Supplementary Figure S5C). These results indicate that ACF1 and WSTF are both involved in TC-NER but not GG-NER, consistent with a function in complex with SMARCA5. Based on these results, we hypothesize that both the ACF and the WICH complex may remodel chromatin during initiation of TC-NER.

GFP-tagged ACF1 and WSTF, stably expressed in U2OS cells, were both recruited to local UV damage (Figure 7A and B). However, their recruitment exhibited a strikingly different accumulation pattern. ACF1-GFP quickly located to the center of the damage spot, after which it spread to the periphery of the damage, similarly as SMARCA5 (Figure 7A). Contrary, GFP-WSTF was even depleted from the center of the damage immediately after damage induction, while it showed a very strong subsequent recruitment to the periphery (Figure 7B). These results implicate both ACF1 and WSTF at sites of UV damage, although with distinct (re)distribution kinetics. To test whether this difference reflects a dynamic interaction with SMARCA5, which may first bind to ACF1 in the center and be handed over to WSTF in the periphery, we performed co-immunoprecipitation after UV. We did not observe any detectable change in each of the different complexes shortly after UV irradiation (Supplementary Figure S5A and B), suggesting that there is no change in the interaction of SMARCA5 with ACF1 or WSTF upon DNA damage induction. Furthermore, we tested whether both subunits could still associate with the SMARCA5 ATPase, HAND and SLIDE domain mutants that show different recruitment patterns (Figure 4). Interestingly, both WSTF and ACF1 co-immunoprecipitated with the ATPase and HAND domain mutants, whereas specifically loss of the SLIDE domain disrupted the interaction of SMARCA5 with ACF1 and WSTF (Figure 7C). These data suggest that the impaired recruitment of the SLIDE domain mutant (Figure 4C) may be related to the inability of SMARCA5 to form complexes with ACF1 and/or WSTF.

Immunoprecipitation of GFP-CSB on MNase-treated nuclear extracts showed that CSB also co-purifies with ACF1 and WSTF (Figure 5C). Furthermore, the recruitment of CSB was attenuated when ACF1 or WSTF was depleted by siRNA (Figure 7D and E), albeit to a lesser extent than for SMARCA5 knock-down (Figure 5A). These findings confirm our results with SMARCA5 and indicate that at least two different ISWI chromatin remodeling complexes, ACF and WICH, function together at sites of DNA damage-stalled transcription to stimulate efficient TC-NER.

## DISCUSSION

Chromatin remodeling during DNA damage repair is thought to be particularly important in regulating the efficiency of lesion recognition (7). Here, we show, in living human cells, that the ATP-dependent chromatin remodeler SMARCA5 facilitates binding of CSB to active TC-NER complexes and controls the repair efficiency of transcription-stalling UV lesions. Furthermore, we show that SMARCA5 binding partners from two distinct ISWI complexes, ACF1 and WSTF, regulate TC-NER initiation in two different discernable kinetic steps. The TC-NER organizing factor CSB also possesses ATP-dependent chromatin remodeling activity (20), and several additional chromatin remodeling proteins were recently implicated in TC-NER and transcription resumption after UV (16–19,22). Together, these observations suggest that extensive chromatin remodeling needs to take place when RNApolII encounters a lesion.

SMARCA5, ACF1 and WSTF function in DSB repair as well (27–29,32), indicating that ISWI is generally important to maintain genome stability. Nevertheless, we identify a novel role for ISWI in TC-NER which is mechanistically distinct from its role in DSB repair, where it stimulates non-homologous end-joining and homologous recombination (28) and promotes the RNF168-mediated ubiquitin signaling response (29). Recruitment and chromatin spreading of SMARCA5 in response to DSBs depends on PARP activity, whereas we did not observe any PARP dependency of SMARCA5 loading and re-localization at sites of UV-C-induced DNA damage. Furthermore, we find that the SMARCA5 ATPase mutant does not localize to UV-C-induced DNA damage, while it does localize to DSBs (28).

**Figure 8.**
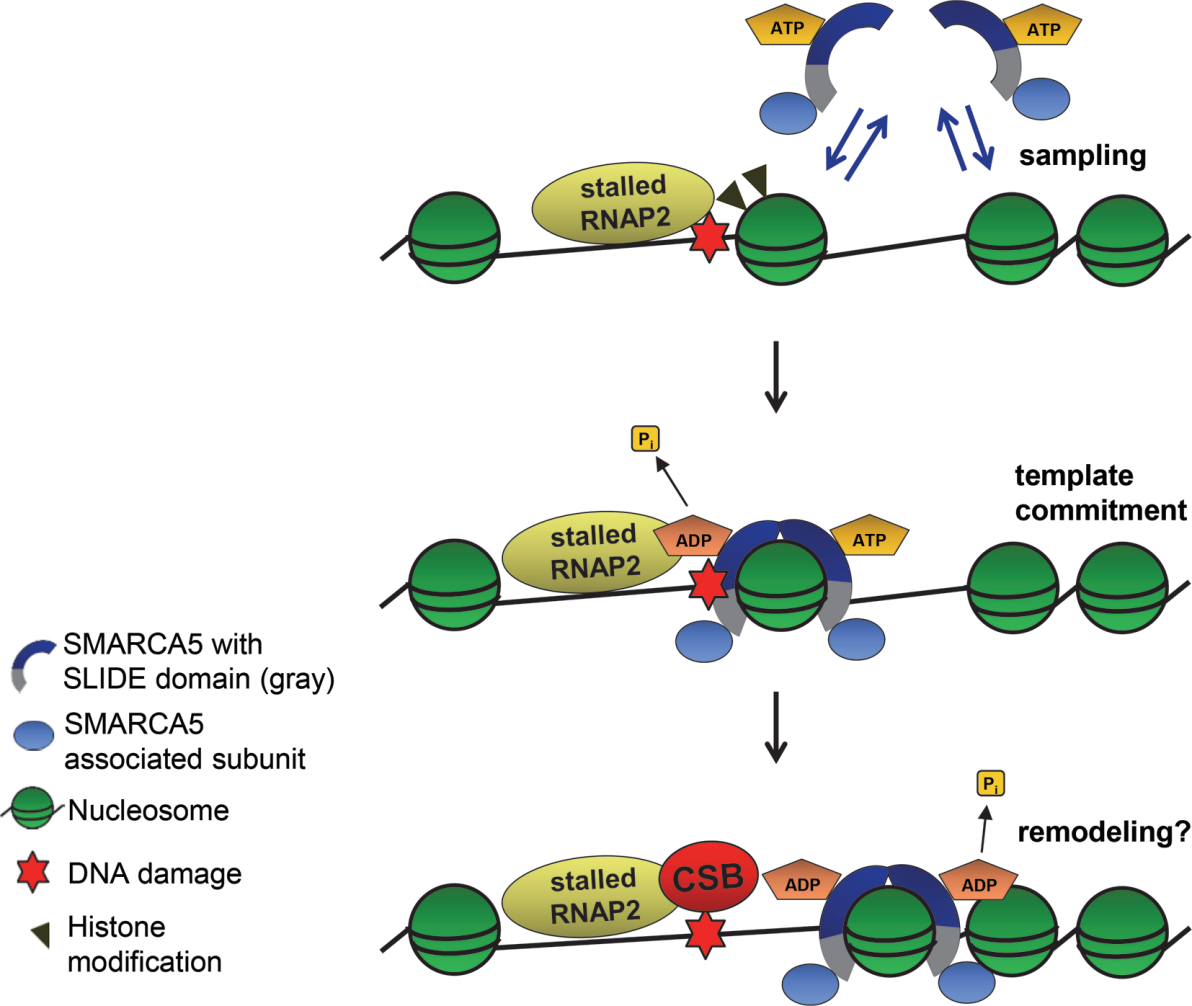
Model for ISWI recruitment and function in TC-NER. SMARCA5 utilizes ATP hydrolysis and its SLIDE domain, which is necessary for the association with ACF1 and WSTF subunits, to scan for and bind to target nucleosomes in the vicinity of lesion-stalled RNApolII. Its recruitment depends on both active transcription and histone modifications. SMARCA5 may remodel chromatin to facilitate efficient CSB association with stalled transcription sites. See the discussion section for details.

Although the exact molecular activity of chromatin remodeling complexes in response to lesion-stalled transcription, including that of ISWI, remains elusive, we do propose a speculative model for SMARCA5 function in this process (Figure 8). ISWI is thought to mediate regular positioning of nucleosomes, especially behind the transcriptional start site of genes (58–60). It is thus conceivable that in TC-NER, ISWI may function to regulate transcriptional activity upon UV damage. Furthermore, both ACF1 and WSTF have been suggested to maintain an open chromatin structure during mammalian replication (61,62). In line with these observations and the reduced CSB recruitment following ISWI knock-down, we propose that the chromatin remodeling capacity of ISWI complexes facilitates an open chromatin structure for efficient CSB association with lesion-stalled transcription complexes. As CSB is necessary for the recruitment of most subsequent TC-NER factors (16), we suggest that chromatin remodeling by ISWI stimulates efficient TC-NER.

The different recruitment kinetics of ACF1 and WSTF to sites of TC-NER (Figure 7A) suggests a functional difference between both subunits in regulating this process. Indeed, different functions for ACF1 and WSTF have been described in literature. In the DSB response, ACF1 was found to interact with and regulate recruitment of Ku70 (28), whereas WSTF was found to interact with and phosphorylate histone H2AX on Tyr142 and to regulate maintenance of Ser139 phosphorylation (32). Moreover, non-catalytic subunits such as ACF1 and WSTF are supposed to regulate the activity and template specificity of the catalytic SMARCA5 subunit, depending on the DNA flanking the nucleosomes (63). Thus, ACF1- and WSTF-containing complexes may have temporally and spatially separated functions during TC-NER as well, to facilitate CSB binding by remodeling chromatin and/or lesion-stalled RNApolII.

Intriguingly, we find that SMARCA5 is first rapidly recruited to the central area of a locally induced DNA damage spot, after which it re-localizes to the damage periphery. This is in striking contrast to CSB and other NER proteins, which accumulate in a concentrated spot. The peripheral re-localization of SMARCA5 may be indicative of actual chromatin remodeling. Surprisingly, deletion of the HAND domain led to a strong initial accumulation of SMARCA5 at local UV damage, but prevented its re-localization to the periphery. Although the function of the SMARCA5 HAND domain is not known, it was postulated to control the directionality of nucleosome sliding due to its contact with the DNA entry/exit site of the nucleosome (64). Thus, it may be that in the absence of the HAND domain, SMARCA5 still associates with nucleosomal targets in damaged chromatin, but its subsequent activity, i.e. chromatin remodeling, is impaired. ACF1 followed a similar initial binding pattern as SMARCA5, but its re-localization to the periphery was less prominent. In contrast, WSTF did not even recruit to the center of damage, but immediately accumulated at the periphery. The initial central localization of SMARCA5 therefore may reflect its association with ACF1, whereas its subsequent peripheral re-localization may reflect its association with WSTF. We tested for a possible handover of SMARCA5 between the different complexes by immunoprecipitation (Supplementary Figure S5A and B), but did not observe a quantifiable change in subunit composition following UV. Although these observations argue against this handover model, it should be noted that the applied procedure, i.e. precipitating the bulk of WSTF- and ACF1-containing complexes, may not be sufficiently sensitive to reveal temporarily changes in composition of only a small fraction of the resident complexes being actively engaged in TC-NER.

Strikingly, SMARCA5 localization to UV-induced DNA damage is independent of NER. It is, however, dependent on transcription, suggesting a direct association of SMARCA5 with lesion-stalled transcription, similar to CSB (36,65) and UVSSA (35). Alternatively, SMARCA5 may continuously scan nucleosomes and bind only to nucleosomal substrates in damaged chromatin, much like XPC scans for DNA damage in GG-NER (66). Previous fluorescence recovery after photobleaching and fluorescence correlation spectroscopy analyses have shown that SMARCA5 complexes are highly mobile and that only a low percentage is transiently bound to chromatin at any given time (67). These findings support a model in which the majority of SMARCA5 molecules continuously sample nucleosomes and only a minor fraction binds to and translocates those nucleosomes that contain a specific cue such as a post-translational modification. Furthermore, ISWI complexes were suggested to use an ATP-hydrolysis-driven kinetic proofreading mechanism to recognize substrate nucleosomes (68,69). Both electron microscopy (70) and single molecule Förster resonance energy transfer studies (71) suggest that ISWI complexes contain dimers of SMARCA5 that first utilize ATP hydrolysis to associate with nucleosomal targets and then utilize a second ATP-hydrolysis event to translocate DNA. Yeast ISW1a and ISW2 chromatin remodeling complexes also bind more stably to nucleosomes depending on ATP hydrolysis (72). Thus, the impaired recruitment of the ATPase-inactive SMARCA5 mutant likely implies that SMARCA5 employs a probing and proofreading mechanism to associate with substrate nucleosomes near damaged DNA in an ATP-hydrolysis-dependent manner (Figure 8).

Besides the ATPase domain, deletion of the SLIDE domain interferes with binding to DNA damage sites. This domain in yeast SMARCA5 orthologs was suggested to help anchor SMARCA5 to the nucleosome through its interaction with extranucleosomal DNA, which facilitates DNA movement into the nucleosome (53,64,73,74). Our results support this idea and indicate that similarly human SMARCA5 utilizes its SLIDE domain, besides ATP hydrolysis, to recognize and bind to nucleosomal targets in the context of damaged DNA. This property of the SLIDE domain may involve ACF1 and WSTF as we show that the SLIDE domain is necessary for the interaction of SMARCA5 with these subunits. The SLIDE-domain-dependent interaction with ACF1 is in agreement with the identification of a small motif at the end of the *Drosophila* ISWI SLIDE domain as the ACF1-interacting domain (75). This same motif is deleted in our human SLIDE mutant and could be necessary for the interaction with WSTF besides ACF1 as well. Furthermore, the above-mentioned ISWI dimerization could explain that in spite of the depletion at the center, ATPase and SLIDE domain mutants still showed slight recruitment to the periphery, as these mutants may still dimerize and travel with functional, endogenous SMARCA5. Conversely, it could be that the ATPase and/or SLIDE mutants are not able to dimerize anymore and therefore show defective DNA damage localization.

The transcription-dependent SMARCA5 translocation to DNA damage suggests that this chromatin remodeler, while continuously probing chromatin, is recruited to a cue that is both DNA damage and transcription dependent. Chromatin targeting and activity of ISWI complexes was shown to depend on histone modifications, such as di- and trimethylation of H3 lysine 4 (51) and hypo-acetylation of the H4 tail (48–50,72,76). Importantly, H4 acetylation levels decrease after UV damage (17). In accordance with these findings, we show that inhibition of histone methyltransferase and deacetylase activity interferes with SMARCA5 binding to DNA damage sites. Therefore, we propose a model in which ISWI chromatin remodeling complexes accumulate at sites of UV damage early during TC-NER, in an ATP-hydrolysis and transcription-dependent manner, which is further stimulated by post-translational histone modifications (Figure 8). Most likely this recruitment results in chromatin remodeling to facilitate efficient CSB recruitment.

Several other ATP-dependent chromatin remodeling factors, i.e. SWI/SNF (9,10), INO80 (14) and ALC1 (33), were recently implicated in mammalian GG-NER. The specificity of the ISWI ATP-dependent chromatin remodeling complexes for TC-NER suggests that specific chromatin configurations characteristic for either GG- or TC-NER requires alternative types of chromatin remodeling events.

## SUPPLEMENTARY DATA

Supplementary Data are available at NAR Online.

SUPPLEMENTARY DATA
