# Supplementary material for: Human ISWI complexes are targeted by SMARCA5 ATPase and SLIDE domains to help resolve lesion-stalled transcription
Source: Nucleic Acids Res. 2014 Jul 2;42(13):8473–85. doi: 10.1093/nar/gku565 (PMC4117783; doi:10.1093/nar/gku565)
Supplement: SUPPLEMENTARY DATA [file supp_42_13_8473__index.html]

Human ISWI complexes are targeted by SMARCA5 ATPase and SLIDE domains to help resolve lesion-stalled transcription — SUPPLEMENTARY DATA 

# Human ISWI complexes are targeted by SMARCA5 ATPase and SLIDE domains to help resolve lesion-stalled transcription

## SUPPLEMENTARY DATA

**Files in this Data Supplement:**

- SUPPLEMENTARY DATA
- SUPPLEMENTARY DATA
- SUPPLEMENTARY DATA
