# Supplementary material for: Human ISWI complexes are targeted by SMARCA5 ATPase and SLIDE domains to help resolve lesion-stalled transcription
Source: Nucleic Acids Res. 2014 Jul 2;42(13):8473–85. doi: 10.1093/nar/gku565 (PMC4117783; doi:10.1093/nar/gku565)
Supplement: SUPPLEMENTARY DATA [file supp_gku565_nar-00549-m-2014-File010.pdf]

Supplementary figure legends

Figure S1. SMARCA5 functions in the transcription-coupled UV response. (A) RRS levels determined by EU incorporation 16 hours after 8 J/m^2 UV in HeLa cells stably expressing shRNA against SMARCA5 show that loss of SMARCA5 leads to reduced recovery of RNA synthesis after UV irradiation. (B) Fluorescence intensity of incorporated EU in SMARCA5 siRNA treated cells is comparable to that of control siRNA treated cells, showing that global transcription levels in non-challenged cells are not affected by SMARCA5 knockdown.

Figure S2. SMARCA5 recruitment to UV-C induced DNA damage. (A) Images (left) show live cell recruitment of SMARCA5-GFP in MRC5 cells to UV-C (266 nm) laser-induced DNA damage (arrows). Graph (right) depicts the normalized fluorescence intensities (n>10 cells) that indicate recruitment at the damage center (blue), the damage periphery (orange) and outside the damaged area (red; mean ± standard error of the mean). (B) Recruitment of SMARCA5 to UV-C induced DNA damage is dose dependent. Normalized SMARCA5-GFP fluorescence intensities at the damage periphery in cells exposed to different UV-C (266 nm) laser intensities: 1% or 10% laser power for 5 seconds (1x) and 10% laser power for 20 seconds (4x; mean ± standard error of the mean, n > 10 cells). RF is Relative Fluorescence. (C) Recruitment of SMARCA5-GFP to local damage induced by irradiation (100 J/m^2) through a microporous filter with a 254 nm UV-C lamp. U2OS cells were fixed 15 minutes and 1 hour after UV. Immunofluorescence staining was performed using antibodies against CPD (red) and SMARCA5 (green). SMARCA5-GFP clearly accumulated at local DNA damage 15 minutes after the damage but 60 minutes after UV irradiation, it was hardly detectable.

Figure S3. Efficient SMARCA5 recruitment depends transcription and shows a dynamic re-localization to the periphery. (A) Graph of the normalized fluorescence intensity indicating recruitment of SMARCA5-GFP to the center (p=0.029 compared to control) and periphery (p=0.114 compared to control) of a UV-C laser-induced DNA damage area in U2OS cells in which transcription is inhibited with DRB (n > 10 cells, mean ± standard error of the mean). RF is Relative Fluorescence. (B) An example cell with SMARCA5-GFP accumulation at local 266 nm UV-induced DNA damage. In the right image, the ‘center’, ‘periphery’ and ‘outside’ regions of interest (ROIs) are indicated that are used to quantify the recruitment of SMARCA5. (C) Co-expression an co-localization of GFP-CSB or SMARCA5-GFP with CPD-Photolyase-mCherry in U2OS cells after local UV-C laser irradiation. SMARCA5 is localized to the periphery of the DNA damage spot indicated by the presence of CPD-photolyase. Scale bar, 5 µm.

Figure S4. SMARCA5 recruitment does not depend on PARP or NER activity. (A) Graph of the normalized fluorescence intensity indicating recruitment of SMARCA5-GFP to the center (p=0.480 compared to control) and periphery (p=0.314 compared to control) of a UV-C laser-induced DNA damage area in U2OS cells treated with PARP inhibitor PJ34 (n > 10 cells, mean ± standard error of the mean). RF is Relative Fluorescence. (B) Immunofluorescence of PAR shows that 5 minutes

treatment with 50 mM H_2O_2 induces PARylation in the nucleus, which is inhibited by treatment with PARP inhibitors Olaparib and PJ34, demonstrating the effectiveness of both inhibitors. Scale bar is 5 µm. (C) SMARCA5-GFP recruitment to UV-C induced DNA damage is normal in CSB-deficient CS1AN fibroblasts, UVSSA-deficient TA24 and XPC-deficient XP4PA fibroblasts. (D) The SMARCA5 SLIDE domain tagged to GFP shows a very weak accumulation signal at UV-C induced DNA damage which disappears within 2 minutes. Scale bar is 5 µm.

Figure S5. ACF1 and WSTF interaction with SMARCA5 does not change after UV. GFP immunoprecipitation of ACF1-GFP (A) and GFP-WSTF (B) in MNase treated nuclear extracts shows that ACF1 and WSTF co-purify with SMARCA5 but not with each other. Immunoprecipitations were performed in unchallenged conditions (-UV) and 5 min after UV irradiation (+UV), showing that the interaction with SMARCA5 does not change upon UV-induced DNA damage induction. (C) Immunoblots showing reduced ACF1 and WSTF expression in either U2OS cells treated with two different siRNAs against ACF1 or an siRNA against WSTF, and in HeLa cells stably expressing an shRNA against WSTF. Tubulin is used as loading control.

Movie S1. GFP-CSB localization to local UV-C DNA damage. The movie depicts the accumulation of GFP-CSB (green) stably expressed in CS1AN cells at local DNA damage induced with a UV-C (266 nm) laser. Images were analyzed by time-lapse confocal microscopy using a laser-scanning confocal microscope (Leica TCS SP5 microscope). The first 14 frames were taken every 5.2 sec, afterwards frames were taken every 10 sec. Frame rate for this video is 3 per second. Total duration is 170 sec.

Movie S2. SMARCA5-GFP accumulation and redistribution upon local UV-C DNA damage induction. The movie depicts the accumulation of SMARCA5-GFP (green) stably expressed in U2OS cells at local DNA damage induced with a UV-C (266 nm) laser. Images were analyzed by time-lapse confocal microscopy using a laser-scanning confocal microscope (Leica TCS SP5 microscope). The first 14 frames were taken every 5.2 sec, afterwards frames were taken every 10 sec. Frame rate for this video is 3 per second. Total duration is 170 sec.

Figure S1

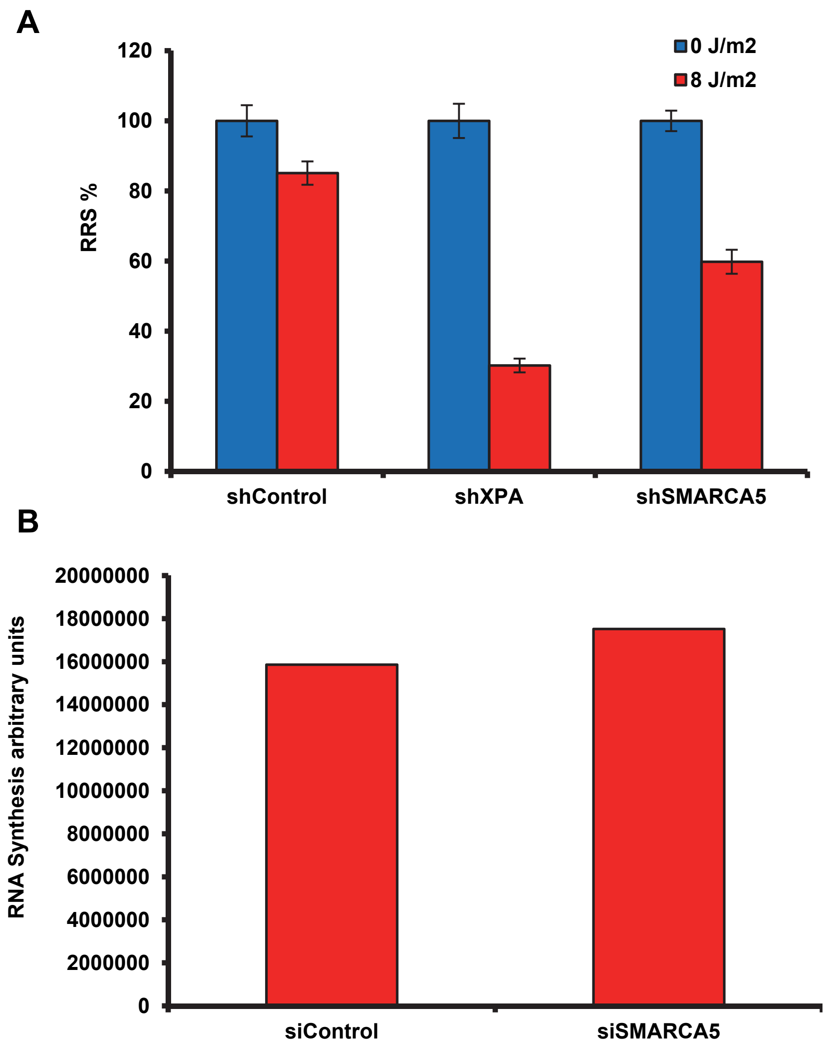
A
0 J/m2
8 J/m2
120
100
80
60
40
20
0
RRS %
shControl
shXPA
shSMARCA5
B
20000000
18000000
16000000
14000000
12000000
10000000
8000000
6000000
4000000
2000000
0
RNA Synthesis arbitrary units
siControl
siSMARCA5

Figure S2

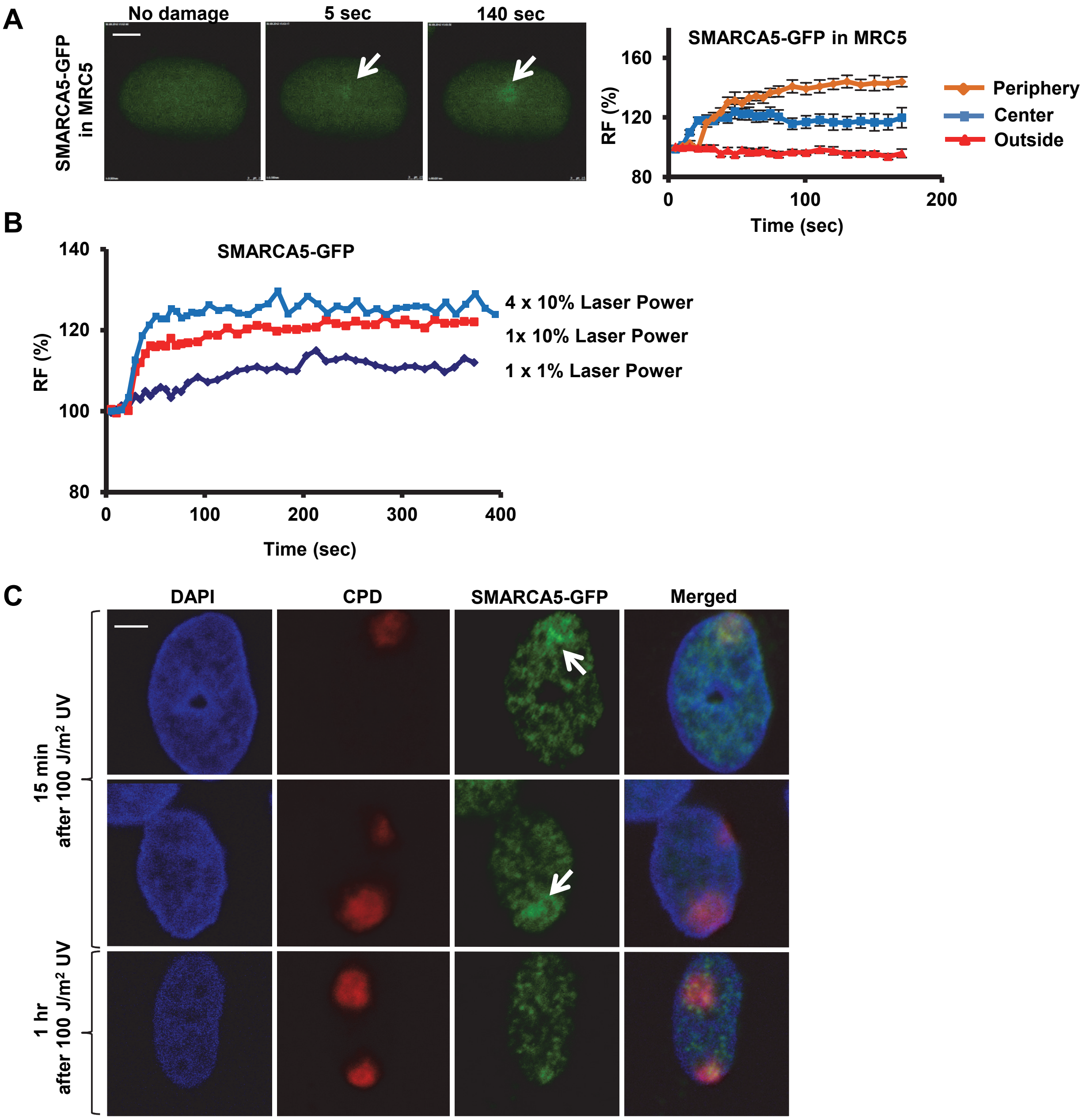

A
No damage
5 sec
140 sec
SMARCA5-GFP in MRC5
SMARCA5-GFP in MRC5
RF (%)
160
120
80
0
100
200
Time (sec)
Periphery
Center
Outside
B
SMARCA5-GFP
140
120
100
80
RF (%)
0
100
200
300
400
Time (sec)
4 x 10% Laser Power
1x 10% Laser Power
1 x 1% Laser Power
C
DAPI
CPD
SMARCA5-GFP
Merged
15 min after 100 J/m² UV
1 hr after 100 J/m² UV

Figure S3

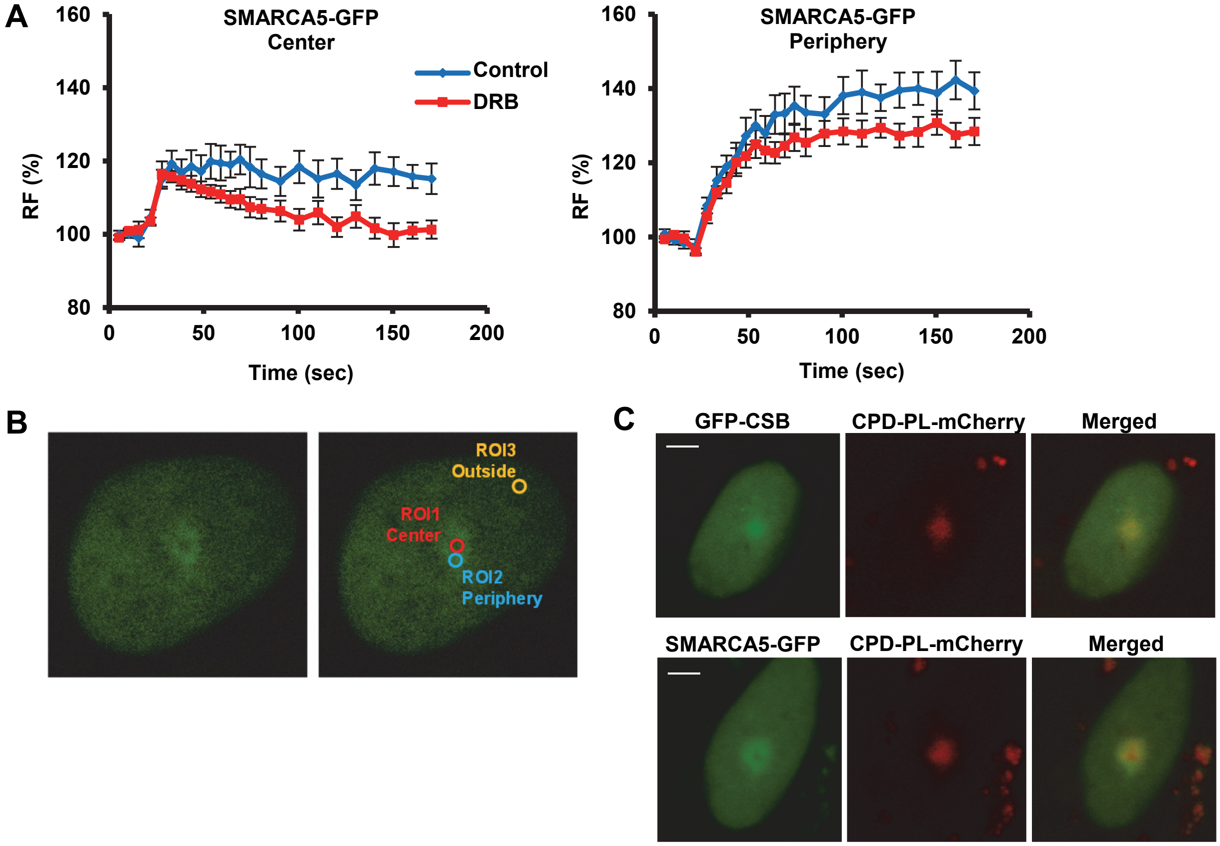
A
SMARCA5-GFP
Center
Control
DRB
RF (%)
160
140
120
100
80
0
50
100
150
200
Time (sec)
SMARCA5-GFP
Periphery
B
ROI3
Outside
ROI1
Center
ROI2
Periphery
C
GFP-CSB
CPD-PL-mCherry
Merged
SMARCA5-GFP
CPD-PL-mCherry
Merged

Figure S4

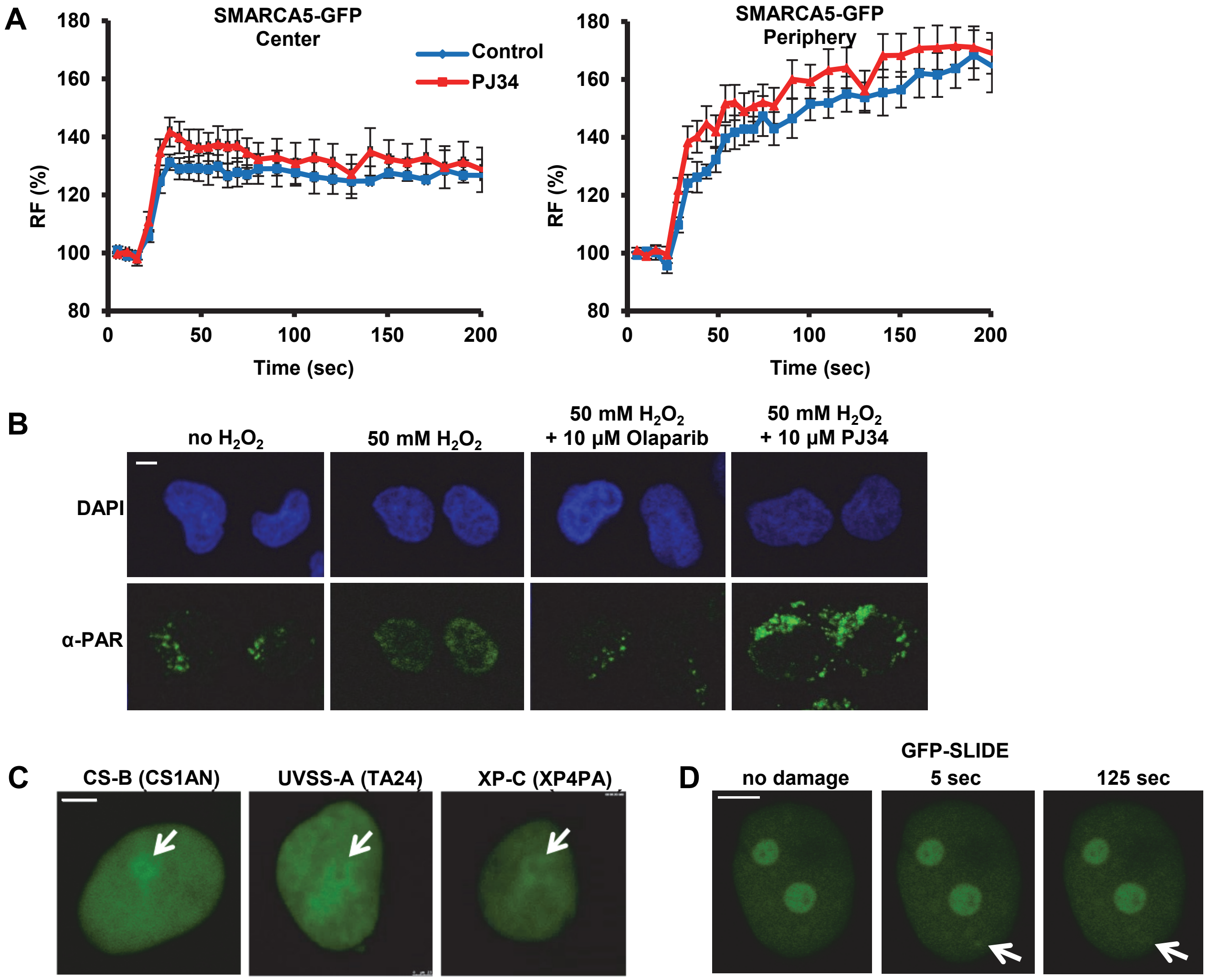
A
SMARCA5-GFP
Center
Control
PJ34
RF (%)
Time (sec)
SMARCA5-GFP
Periphery
B
no H_2O_2
50 mM H_2O_2
50 mM H_2O_2 + 10 μM Olaparib
50 mM H_2O_2 + 10 μM PJ34
DAPI
α-PAR
C
CS-B (CS1AN)
UVSS-A (TA24)
XP-C (XP4PA)
D
GFP-SLIDE
no damage
5 sec
125 sec

Figure S5

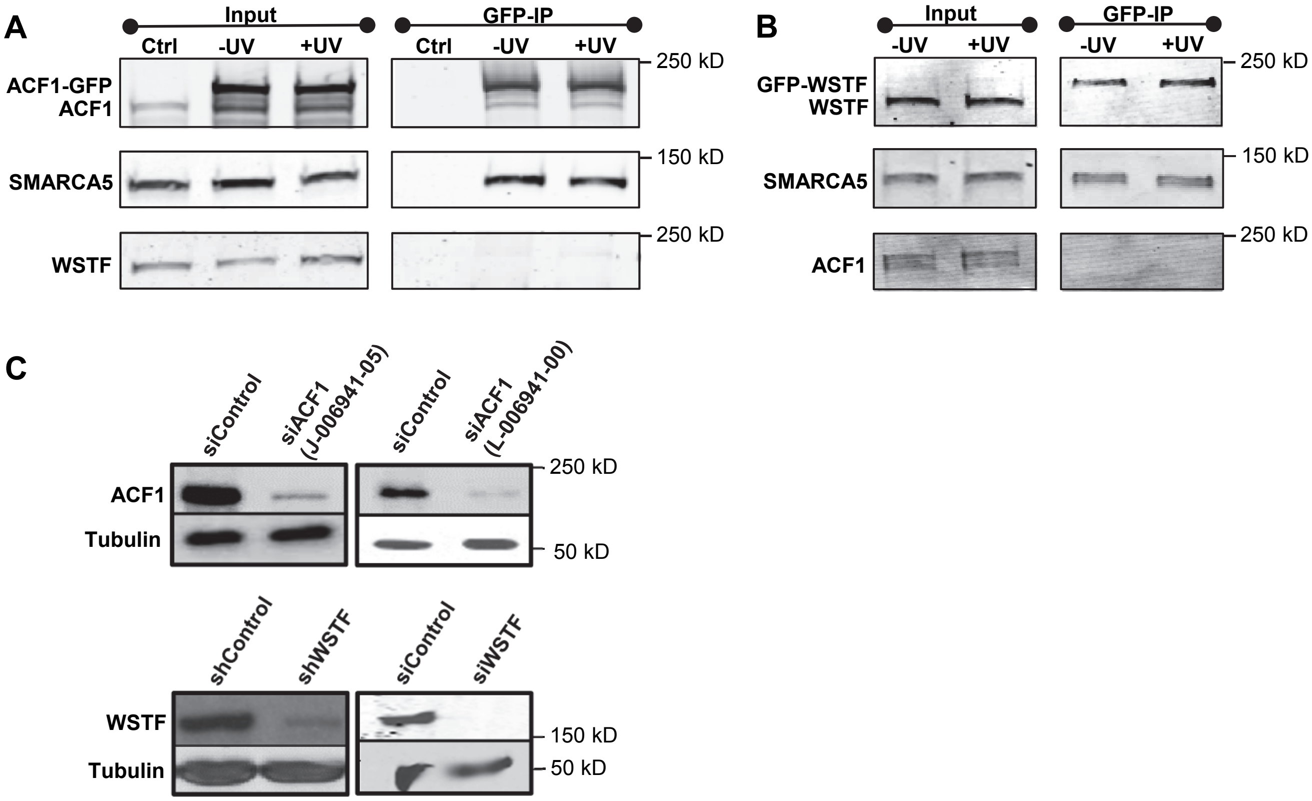
A
Input
GFP-IP
Ctrl
-UV
+UV
ACF1-GFP
ACF1
SMARCA5
WSTF
250 kD
150 kD
250 kD
B
Input
GFP-IP
-UV
+UV
GFP-WSTF
WSTF
SMARCA5
ACF1
250 kD
150 kD
250 kD
C
siControl
siACF1 (J-006941-05)
siControl
siACF1 (L-006941-00)
ACF1
Tubulin
250 kD
50 kD
shControl
shWSTF
siControl
siWSTF
WSTF
Tubulin
150 kD
50 kD
